# CircATP2C1 Drives Prostate Cancer Progression Through miR-654-3p-Mediated SLC7A11 Upregulation and Ferroptosis Suppression

**DOI:** 10.3390/cancers17213571

**Published:** 2025-11-05

**Authors:** Zhihai Deng, Qiang Shen, Nan Deng, Jun Wu, Xinghui Cheng, Jiaxing Wang, Hangyang Peng, Weijie Zeng, Ziyi Song, Dongmei Jiang, Daojun Lv, Xiangming Mao

**Affiliations:** 1 Department of Urology, Zhujiang Hospital, Southern Medical University, Guangzhou 510280, China; d985840071@smu.edu.cn (Z.D.); junwu1998y@163.com (J.W.); 18787426659@163.com (J.W.); phy2992@163.com (H.P.); 2 Department of Urology, Guangdong Provincial Key Laboratory of Major Obstetric Diseases, Guangdong Provincial Clinical Research Center for Obstetrics and Gynecology, The Third Affiliated Hospital, Guangzhou Medical University, Guangzhou 510150, China; shenqiang7972@163.com (Q.S.); dannis2004@163.com (N.D.); 2023111388@stu.gzhmu.edu.cn (X.C.); 15622157308@163.com (W.Z.); k131724@163.com (Z.S.); 3 Department of Pathology, The First Affiliated Hospital of Guangzhou Medical University, 151 West Yanjiang Road, Guangzhou 510120, China; 2018681116@gzhmu.edu.cn

**Keywords:** prostate cancer, CircATP2C1, ferroptosis, miR-654-3p, SLC7A11

## Abstract

**Background**: Prostate cancer, an epithelial malignancy occurring in the prostate, is the most common malignant tumor of the male genitourinary system and has a low survival rate in advanced prostate cancer after metastasis. It is urgent to explore novel therapeutic targets and strategies for treating prostate cancer. Circular RNA (circRNA) and ferroptosis both play critical roles in prostate cancer progression. However, the regulatory effect of circRNA on ferroptosis remains unclear. **Methods**: Here, circRNA expression profiles in prostate cancer were explored by bioinformatics analysis and human prostate cancer tissue microarray. Stable circRNA-knockdown or overexpressed prostate cancer cell lines were constructed by lentivirus. AGO2-RNA immunoprecipitation (AGO2-RIP) was utilized to identify circRNA-microRNA (miRNA) interaction. **Results**: Results of this study indicate that circATP2C1 is highly expressed in prostate cancer. In addition, circATP2C1 promotes prostate cancer cell proliferation, migration, and invasion by suppressing ferroptosis in vitro. Moreover, circATP2C1 facilitates the tumorigenicity of prostate cancer by inhibiting ferroptosis in vivo. **Conclusions**: Mechanistically, circATP2C1 hinders ferroptosis by increasing solute carrier family 7 member 11 (SLC7A11) expression via sponging miR-654-3p. In summary, our findings highlight the oncogenic role of circATP2C1 in prostate cancer and provide novel targets and strategies for treating prostate cancer.

## Simple Summary

Prostate cancer (PCa) remains a leading cause of male cancer mortality globally. Biomarker discovery—from liquid biopsies (ctDNA and CTCs) to novel imaging (PSMA-PET)—aims to improve early diagnosis, risk stratification (using genomic classifiers), and therapy response monitoring. Firstly, we validated, through 60 pairs of prostate cancer tissue microarrays, that circATP2C1 is highly expressed in prostate cancer tissues and is positively correlated with TNM staging, suggesting its potential as a predictive biomarker for prostate cancer. Secondly, in vitro and in vivo experiments confirmed that circATP2C1 promotes the proliferation and migration of prostate cancer cells. Furthermore, the combined treatment of circATP2C1 knockdown and erastin significantly inhibited tumor cell proliferation and migration, as well as suppressed prostate cancer growth; our findings highlight the oncogenic role of circATP2C1 in prostate cancer and provide novel targets and strategies for treating prostate cancer.

## 1. Introduction

The latest global cancer statistics indicate that prostate cancer is gradually becoming the most common cancer and the second leading cause of cancer death in men [1]. Prostate cancer is a relatively indolent cancer, and the 5-year survival rate of localized prostate cancer can reach 97%. However, for advanced metastatic prostate cancer, the 5-year survival rate is less than 30%, showing a substantial difference in prognosis [2]. More than 50% of prostate cancer patients in China have already experienced local progression or metastasis at the time of diagnosis, which is a clinical problem that requires urgent attention and solution. In recent years, circular RNA (circRNA), as a novel regulatory molecule in epigenetics, has provided a new direction to reveal the mechanism of tumor disease occurrence and progression [3]. Increasing evidence shows that circRNA plays a crucial role in the proliferation, apoptosis, and metastasis of tumor cells, including prostate cancer [4,5,6,7], lung cancer [8], breast cancer [9], gastric cancer [10], liver cancer [11], and colon cancer [12]. As a competitive endogenous RNA (ceRNA), circRNA can competitively bind miRNAs, thereby regulating the expression of miRNA target genes [13]. For example, circ-ASH2L can act as a ceRNA for miR-34a and activate the Notch-1 pathway by regulating miR-34a expression, promoting tumor invasion, proliferation, and angiogenesis [14]. Circ_0092314 can act as a sponge for miR-671, leading to increased expression of S100P protein, which in turn induces epithelial–mesenchymal transition in pancreatic cancer cells and promotes pancreatic cancer development [15]. However, the precise mechanism of circRNAs regulating prostate cancer needs further exploration [16,17,18].

Ferroptosis is a newly discovered type of cell death dependent on Fe2+ overload and lipid peroxidation, which is morphologically, physiologically, and biochemically distinct from classical programmed cell death, apoptosis, or pyroptosis [19]. It has been found that there is crosstalk between ferroptosis and a number of classical cellular signals in tumors, such as lactic acid produced by tumor metabolism. In addition to contributing to the formation of a microenvironment prone to metastasis, lactic acid acts through the hydroxy-carboxylic acid receptor 1 (HCAR1), monocarboxylate transporter 1 (MCT1), and sterol regulatory element binding protein 1 (SREBP1) pathway. The SREBP1 and stearoyl coenzyme A desaturase 1 (SCD1) pathway inhibits ferroptosis in tumor cells, leading to tumor progression and metastasis [20]. Recently, it has been shown that neighboring cells can also activate the NF2 and Hippo signaling pathways through E-cadherin-mediated interactions to inhibit ferroptosis in tumor cells [21]. In addition, solute carrier family 7 member 11 (SLC7A11) can inhibit cellular ferroptosis by cystine uptake, promoting glutathione (GSH) synthesis, and preventing the accumulation of lipid peroxidation products [22,23,24,25]. Thus, ferroptosis regulates tumor cell growth and metastasis. Nevertheless, circRNAs modulating SLC7A11 in prostate cancer remain unclear [26,27,28].

Our study indicates that circATP2C1 is highly expressed in prostate cancer, especially in highly metastatic and rapidly proliferating prostate cancer cells. Results of high-throughput sequencing reveal that knockdown of circATP2C1 leads to alterations in the ferroptosis pathway. Based on these findings, we hypothesize that circATP2C1 may modulate the proliferation and metastasis of prostate cancer cells through the regulation of ferroptosis.

## 2. Materials and Methods

### 2.1. Bioinformatics Analysis

Gene expression profiles of circRNAs in prostate cancer were obtained from the Gene Expression Omnibus (GEO, Boca Raton, FL, USA) DataSets (GSE179321 and GSE155792) of the National Center for Biotechnology Information (NCBI, Bethesda, MD, USA). In addition, potential targeted miRNAs of circATP2C1 were identified by Circbank, Circbase, Starbase, and Circinteractome databases. Targetscan, miRwalk, and Starbase were used to predict the target genes of miR-654-3p.

### 2.2. Cell Culture

The normal human prostate cell line RWPE-1 and prostate cancer cell lines (C4-2, 22RV1, DU145, LNCaP, and PC3) were obtained from the Cell Bank at the Chinese Academy of Sciences (China). RWPE-1 cells were cultured in keratinocyte serum-free medium (Shanghai QiDa Biotechnology, Shanghai China) supplemented with 50 μg/mL bovine pituitary extract and 5ng/mL epidermal growth factor. C4-2, 22RV1, DU145, LNCaP, and PC3 cells were maintained in RPMI 1640 (Gibco, NewYork, NY, USA) containing 10% fetal bovine serum (FBS, HyClone, USA). All cells were grown in a humidified atmosphere with 5% CO_2_ at 37 °C.

### 2.3. Quantitative Reverse Transcription-PCR (qRT-PCR)

Based on the primers (Table 1) designed for each gene. Total RNA was extracted from cells using TRIzol (Invitrogen, Carlsbad, CA, USA). For extracting circRNAs, RNase R was used to treat total RNA to remove linear RNAs. First-strand cDNA was made by PrimeScript II 1st Strand cDNA Synthesis Kit (Takara Biotechnology, Dalian, China), and then, qRT-PCR was performed using TB Green Premix Ex Taq (Tli RNaseH Plus) (Takara Biotechnology). Results of target RNAs were normalized to that of GAPDH and presented as 2^(−ΔΔCt) values relative to the control group.

**Table 1:** Sequences of primers used for qRT-PCR.

Genes		Sequences (5’-3’)
**Hsa_circ_ATP2C1**	Forward	GCAGACATGATCCTAGTGGATG
	Reverse	GCTGGGTTATAGAATCCATGAAC
**ATP2C1**	Forward	CTGGTCCTGAACTGGGACAGC
	Reverse	CGATTGCAACTGCAGTCTCC
**miR-654-3p**	Forward	acactccagctgggTATGTCTGCTGACCATCA
	Reverse	CTCCATCTTGCCTCTTGGCC
**GPX4**	Forward	GGGCTACAACGTCAAATTCG
	Reverse	TCGATGAGGAACTTGGTGAA
**SLC7A11**	Forward	CTCTGGCACTGTGATCATGAA
	Reverse	CTCCCCAGAGGGAACTCATTT
**GAPDH**	Forward	CATCACTGCCACCCAGAAGAC
	Reverse	GAGCTTCCCGTTCAGCTCAG
**U6**	Forward	TCGCTTCGGCAGCACA
	Reverse	ACGCTTCACGAATTTGCG

### 2.4. Human Prostate Cancer Tissue Microarray

Human prostate cancer tissue microarray was purchased from Shanghai Outdo Biotech (Shanghai, China) and used to analyse the relationship between circATP2C1 expression and survival, Gleason score (GS), migratory capacity, LNM stage, and recurrence rate of patients with prostate cancer. Clinicopathologic characteristics of the human prostate cancer tissue microarray are listed in Supplementary Table S1.

### 2.5. Cell Treatment

RNase R and actinomycin D were used to characterize circRNA in prostate cancer cells. Briefly, 4 µg total RNA was incubated with RNase R (4 U/µg RNA, Geneseed Technologies, Guangzhou, China) for 10 min at 37 °C, followed by assessing the relative expression levels of circRNA and mRNA using qRT-PCR. Additionally, 2 mg/mL actinomycin (Sigma-Aldrich, St. Louis, MO, USA) was used to incubate cells, and then cells were harvested at 0 h, 4 h, 8 h, 12 h, and 24 h post-actinomycin treatment to detect expression levels of circRNA and mRNA by qRT-PCR.

### 2.6. Construction of Stable Cell Lines

To construct stable circATP2C1-knockdown PC3 and LNCaP cell lines, short hairpin RNA (shRNA) targeting circATP2C1 was first designed according to small interfering RNAs (siRNAs). Then, two single DNA strands of shRNA targeting circATP2C1 were inserted into the lentivirus interference vector pLVX-shRNA1 to establish the lentivirus shRNA interference vectors for circATP2C1, followed by the co-transfection with lentivirus packaging helper plasmids pLP1 and pLP2 into 293 FT cells to package lentivirus containing shRNA targeting circATP2C1. To establish stable circATP2C1-expressed PC3 and LNCaP cell lines, the circATP2C1 sequence was cloned into the lentivirus expression vector pLV-circ-Puro, followed by the co-transfection with lentivirus packaging helper plasmids pLP1 and pLP2 into 293 FT cells to package lentivirus containing the circATP2C1 expression vector. Subsequently, PC3 and LNCaP cells were maintained in conditioned medium containing lentivirus and 5 μg/mL polybrene for 48 h. Finally, transfected cells were selected by puromycin to construct stable circATP2C1-knockdown (si-circATP2C1) and circATP2C1-expressed (circATP2C1) PC3 and LNCaP cell lines, which were verified using qRT-PCR.

### 2.7. Cell Counting Kit-8 (CCK-8) Assay

PC3 and LNCaP cells were placed in a 96-well plate and then incubated with CCK-8 solution (Beyotime, Shanghai, China) for 2 h at 37 °C. Next, the absorbance at 450 nm of PC3 and LNCaP cells was assayed using the Multiscan MK3 microreader (Thermo Fisher Scientific, Waltham, MA, USA).

### 2.8. Transwell Assay for Cell Invasion and Migration

In the Transwell assay for invasion, 50 mL Matrigel (BD Biosciences, Franklin Lakes, NJ, USA) was coated on the inner side of the 8 μm pore polycarbonate membrane inserts of Transwell chambers (BD Biosciences). After starvation, PC3 and LNCaP cells were placed into the upper chambers. 48 h later, Transwell chambers were fixed with 4% paraformaldehyde (PFA) for 30 min at 25 °C. Then, crystal violet was used to dye cells in the lower chambers for 30 min at 25 °C. Subsequently, the number of crystal violet-dyed cells was counted under a microscope.

### 2.9. Scratch Assay

Originally, PC3 and LNCaP cells were seeded in a six-well plate for 24 h. Then, a straight line across the plate was drawn by a pipette tip. Next, RPMI 1640 medium containing 2% FBS was added to incubate the cells. Subsequently, images of scratched cells were captured after 0 h, 12 h, 24 h, 36 h, and 48 h by an inverted optical microscope. The migrative ability of cells was identified according to the healed area of the scratch.

### 2.10. RNA Sequencing

Initially, total RNA was extracted from LNCaP cells by TRIzol (Invitrogen), and then cDNA libraries were established and sequenced by Illumina NextSeq 500. Next, clean reads with high quality were collected, followed by the calculation of mRNA levels to identify differentially expressed genes (DEGs) in circATP2C1-knockdown LNCaP cells compared to control LNCaP cells. Subsequently, DEGs were mapped according to Gene Ontology (GO) terms, and enriched pathways in DEGs were identified using KEGG and Gene Set Enrichment Analysis (GSEA).

### 2.11. ELISA

Ferroptosis-related indicators malondialdehyde (MDA), Fe2+, and glutathione (GSH) were detected by MDA(Malondialdehyde) ELISA Kit (FineTest, Wuhan,  China), Human Fe2+ ELISA Kit (Shanghai Win-Win Biotechnology, Shanghai, China), and Human GSH ELISA Kit (Shanghai Zhenke Biology, Shanghai China) according to the manufacturer’s instructions, respectively.

### 2.12. Electron Microscopy

Mitochondrial morphology of PC3 and LNCaP cells was observed by electron microscopy. Briefly, PC3 and LNCaP cells were first fixed by 2% PFA containing 0.2% glutaraldehyde for 1 h at 37 °C, followed by dehydration via a graded ethanol series. Then, PC3 and LNCaP cells were embedded, and 75 nm ultrathin sections of PC3 and LNCaP cells were mounted on nickel grids. Next, nickel grids were stained and visualized using an electron microscope at 80 kV.

### 2.13. FISH

FITC-labeled antisense and sense probes targeting circATP2C1 were synthesized, and then, FISH was conducted on PC3 and LNCaP cells to detect circATP2C1 location following the established protocol.

### 2.14. Dual Luciferase Reporter Assay

Initially, the WT or MT sequence of the miR-654-3p binding site in circATP2C1 or SLC7A11 mRNA 3′UTR was cloned into the luciferase reporter gene vectors, which were co-transfected with mimic NC and miR-654-3p mimic, respectively, into 293T cells. Then, 293T cells were subjected to luciferase activity determination by the Dual Luciferase Reporter Assay System (Promega, Madison, WI, USA) at 48 h after co-transfection.

### 2.15. AGO2-RIP

PC3 and LNCaP cells were lysed, and then, nucleic acid fragments were disrupted using ultrasound. Next, the cell lysate was incubated with AGO2 antibody (1:50, #ab186733, Abcam, Cambridge, UK) overnight at 4 °C. Subsequently, AGO2-binding RNA fragments were captured using avidin magnetic beads and identified by qRT-PCR.

### 2.16. WB

To isolate total proteins, PC3 and LNCaP cells were incubated with 200 μL RIPA lysis buffer (Beyotime) for 15 min at 4 °C. Then, PC3 and LNCaP cells were centrifuged at 14,000 rpm for 15 min at 4 °C to gain total proteins. Proteins from PC3 and LNCaP cells were separated by electrophoresis into SDS-polyacrylamide gel followed by the transfer onto PVDF membranes (Millipore, Burlington, MA, USA). Membranes were blocked utilizing 5% non-fat milk for 1 h at 25 °C and then incubated with primary antibodies overnight at 4 °C. Next, membranes were washed, followed by the incubation with secondary antibodies for 1 h at 25 °C. Finally, the Chemiluminescence Detection Kit (Beyotime) was used to visualize targeted protein signals. Densitometry of target protein bands was qualified. The primary antibodies used for WB included SLC7A11 antibody (1:1000, #ab307601, Abcam), Glutathione Peroxidase 4 (GPX4) antibody (1:1000, #ab125066, Abcam) and GAPDH antibody (1:10,000, #KC-5G5, Aksomics, Shanghai,  China).

### 2.17. In Vivo Assay

Animal experiments were approved by the Institutional Animal Care and Use Committee of Zhujiang Hospital, Southern Medical University (LAEC-2024-253). Initially, four-week-old male BALB/c nude mice were raised in a pathogen-free environment. Then, xenograft tumor models were constructed by subcutaneous injection of PC3 cells into the right dorsal flanks of nude mice. In addition, erastin was administered to the nude mice by intragastric administration on Day 7 after subcutaneous injections of PC3 cells. Subsequently, the size of the xenograft tumors was measured every three days. Finally, mice were sacrificed at Day 29 post subcutaneous injection of PC3 cells, then xenograft tumors were harvested, and the weight of the tumors was identified.

### 2.18. Immunohistochemistry (IHC)

Tumor tissues were collected from mice and fixed with 4% PFA, embedded, and sliced. After deparaffinization and rehydration, sections of tumor tissues were permeabilized by 0.2% Triton X-100 dissolved in PBS for 1 h at 25 °C and blocked with 1% BSA and 5% goat serum in PBS containing 0.1% Triton X-100 for 1 h. Then sections were incubated with SLC7A11 antibody (1:500, #ab307601, Abcam) overnight at 4 °C. The next day, sections were washed and incubated with the biotin-labeled secondary antibody for 1 h at 25 °C. Subsequently, sections were washed, followed by the incubation with HRP Streptavidin (Yeasen, Shanghai, China) for 30min at 25 °C. Finally, sections were stained by DAB and photographed. The presence of staining artifacts was applied to measure the mean optical density (OD) for SLC7A11.

### 2.19. Statistical Analysis

In this study, quantitative data were presented as mean ± standard deviation (SD). SPSS 20 (SPSS Inc., Chicago, IL, USA) was utilized for the analysis of statistical differences. The unpaired Student’s *t*-test was used for the comparison between two groups. In addition, the post hoc Tukey’s test following one-way ANOVA was utilized for the statistics among multiple groups. *p* < 0.05 was considered statistically significant.

## 3. Results

### 3.1. CircATP2C1 Is Highly Expressed in Prostate Cancer

**Figure 1:**
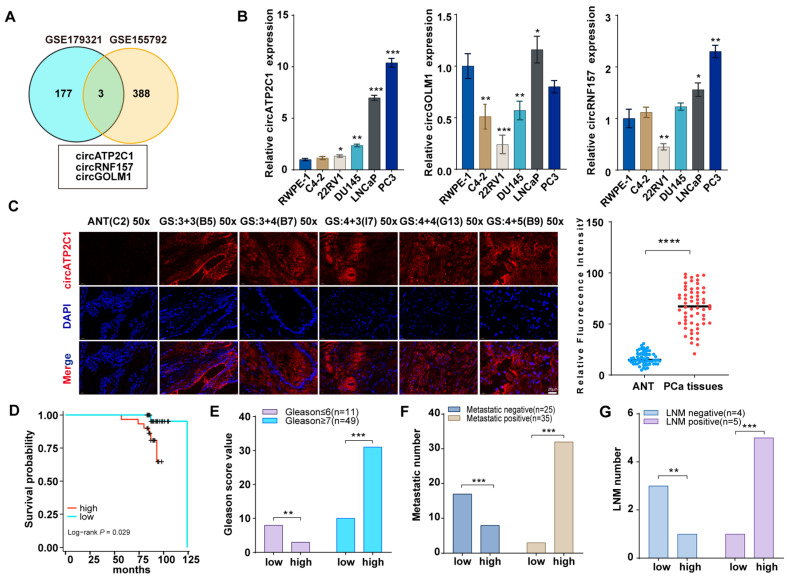
CircATP2C1 is highly expressed in prostate cancer and may be involved in the proliferation and metastasis of prostate cancer. (**A**) Venn diagram illustrating highly expressed circRNAs identified from prostate cancer sequencing data. (**B**) qPCR was performed to detect the expression of circATP2C1, circRNF157, and circGOLM1 in normal prostate epithelial cells and five types of prostate cancer cells. (**C**) The circATP2C1 level in prostate cancer tissues and adjacent non-tumor (ANT) samples was analyzed by FISH. (**D**) Prostate cancer patients were divided into circATP2C1-high and circATP2C1-low expression groups, and the survival of the two groups was analyzed. (**E**) The Gleason score of prostate cancer patients in the circATP2C1-high and circATP2C1-low groups was analyzed. (**F**) The metastatic status of prostate cancer patients in both circATP2C1-high and circATP2C1-low groups was analyzed. (**G**) The LNM status of prostate cancer patients in both circATP2C1-high and circATP2C1-low groups was analyzed. PCa, prostate cancer; ANT, adjacent non-tumor tissue; GS: Gleason score, where * *p* < 0.05, ** *p* < 0.01, *** *p* < 0.001, **** *p* < 0.0001.

Gene expression profiles of circRNAs in prostate cancer were obtained from GEO datasets (GSE179321 and GSE155792, logFC > 2, *p* < 0.05), and further analysis revealed that circATP2C1, circRNF157, and circGOLM1 are highly expressed in prostate cancer (Figure 1A). Then, the levels of circATP2C1, circRNF157, and circGOLM1 were measured in the normal prostate cell line RWPE-1 and the prostate cancer cell lines (C4-2, 22RV1, DU145, LNCaP, and PC3). The results showed that circATP2C1 expression was elevated in metastatic and fast-growing prostate cancer cells (Figure 1B). Moreover, fluorescence in situ hybridization (FISH) results indicated that circATP2C1 levels were significantly increased in prostate cancer tissues compared to adjacent non-tumor tissue (ANT) samples (Figure 1C). Additionally, patients with high circATP2C1 expression in prostate cancer showed lower overall survival, higher Gleason score (GS), greater migratory capacity, and more advanced lymph node metastasis (LNM) stage (Figure 1D–G). These data suggest that circATP2C1 is highly expressed in prostate cancer and may contribute to the cell proliferation and metastasis of prostate cancer cells.

#### 3.1.1. Identification of circATP2C1 in Prostate Cancer Cells

**Figure 2:**
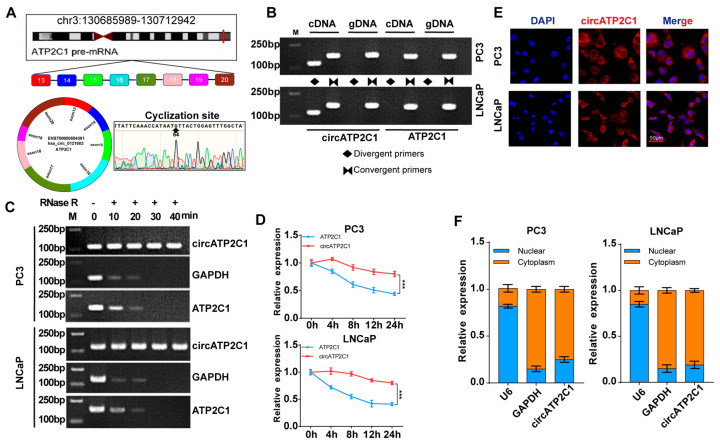
CircATP2C1 is cyclically expressed in the cytoplasm of prostate cancer cells. (**A**) Illustration of the loop formation position of circATP2C1 and corresponding sequencing results. (**B**) PCR detection of circATP2C1 expression in PC3 and LNCaP cells. The rhombus indicates the amplification with divergent primers, while two opposing triangles indicate the amplification with convergent primers. (**C**) qPCR detection of circATP2C1 expression in PC3 and LNCaP cells after treatment with RNase R. (**D**) Detection of ATP2C1 and circATP2C1 by qPCR in PC3 and LNCaP cells after treatment with actinomycin D for 0, 4, 8, 12, 24 h. (**E**) Localization of circATP2C1 was detected by FISH in PC3 and LNCaP cells. (**F**) Following nucleoplasmic separation in PC3 and LNCaP cells, qPCR was performed to detect circATP2C1 expression in the nucleus and cytoplasm. *** *p* < 0.001.

Before testing the effects of circATP2C1 on prostate cancer cells, it was necessary to characterize circATP2C1 in these cells. Sequencing and PCR assay results showed that circATP2C1 formed a circular junction (Figure 2A,B). Moreover, circATP2C1 expression in prostate cancer cells did not change significantly after RNase R treatment, whereas mRNA expressions of linear ATP2C1 and GAPDH were significantly reduced (Figure 2C). Following treatment with actinomycin D for 0 h, 4 h, 8 h, 12 h, and 24 h, the expression level of circATP2C1 was significantly higher than that of ATP2C1 (Figure 2D). These results suggest that circATP2C1 forms a circular RNA and is stably expressed in prostate cancer cells. Moreover, the localization of circATP2C1 in PC3 and LNCaP cells was determined by fluorescence in situ hybridization (FISH) and nucleoplasmic separation. Results suggest that circATP2C1 is mainly localized in the cytoplasm of PC3 and LNCaP cells (Figure 2E,F).

#### 3.1.2. CircATP2C1 Facilitates Prostate Cancer Cell Proliferation, Migration, and Invasion

**Figure 3:**
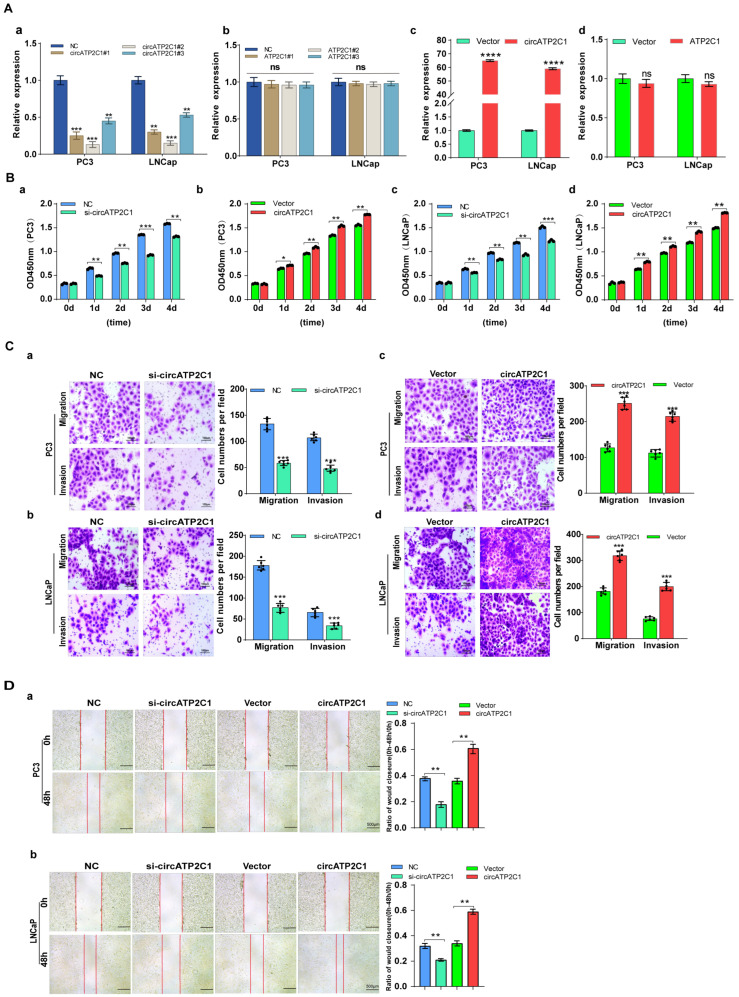
CircATP2C1 facilitates prostate cancer cell proliferation, migration, and invasion. (**A**) In PC3 and LNCaP cells, qPCR was performed to assess the effect of siRNA on the expression of circATP2C1 (**a**) and its host gene ATP2C1 (**b**). Additionally, the effect of transfection with an overexpression plasmid on the expression of circATP2C1 (**c**) and ATP2C1 (**d**) was evaluated. (**B**) Effects of knockdown (**a**,**c**) and overexpression (**b**,**d**) of circATP2C1 on cell proliferation were analyzed using the CCK8 assay in PC3 and LNCaP cells. (**C**) In PC3 and LNCaP cells, Transwell assays were conducted to examine the effects of knockdown (**a**,**b**) and overexpression (**c**,**d**) of circATP2C1 on cell migration and invasion. (**D**) Effects of knockdown and overexpression of circATP2C1 on cell migration were assessed by scratch assay in PC3 (**a**) and LNCaP (**b**) cells.NC, negative control; si-circATP2C1, circATP2C1 knockdown; OE-circATP2C1, circATP2C1 overexpression. * *p* < 0.05, ** *p* < 0.01, *** *p* < 0.001, **** *p* < 0.0001.

Next, the effects of circATP2C1 on prostate cancer cells were investigated. First, siRNAs were designed according to the sequence near the circATP2C1 junction, and the knockdown efficiency of the siRNAs was assessed (Figure 3(Aa)). siRNA2, which showed higher knockdown efficiency, was selected to design shRNA and construct a stable knockdown cell line for prostate cancer cells. Furthermore, knockdown of linear ATP2C1 had no effect on circATP2C1 expression (Figure 3(Ab)). At the same time, a stable circATP2C1 overexpression cell line was constructed. Results from the qPCR assay indicated that the stable overexpression cell lines were constructed successfully (Figure 3(Ac)); however, overexpression of linear ATP2C1 had no effect on circATP2C1 expression (Figure 3(Ad)). Moreover, overexpression of circATP2C1 promoted proliferation, migration, and invasion, whereas knockdown of circATP2C1 suppressed these abilities in both PC3 and LNCaP cells (Figure 3(Ba–d), Figure 3(Ca–d), and Figure 3(Da,b)). These data suggest that circATP2C1 enhances prostate cancer cell proliferation, migration, and invasion.

#### 3.1.3. CircATP2C1 Adsorbs miR-654-3p to Enhance Proliferation, Migration, and Invasion of Prostate Cancer Cells

**Figure 4:**
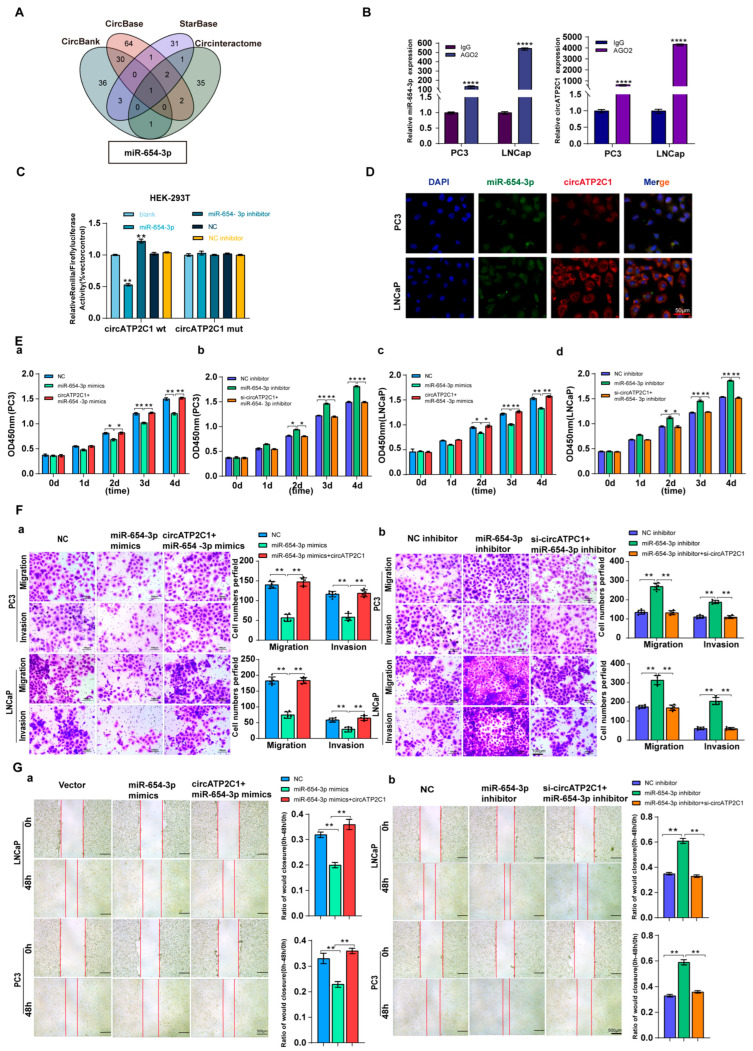
CircATP2C1 adsorbs miR-654-3p to enhance the proliferation, migration, and invasion of prostate cancer cells. (**A**) bioinformatics analysis of circATP2C1-bound microRNAs. (**B**) The binding of miR-654-3p and circATP2C1 was detected by AGO2-RIP-qPCR in human prostate cancer PC3 and LNCaP cells. (**C**) Dual-luciferase assay detecting the binding between miR-654-3p and circATP2C1 in the 293T cells. (**D**) Co-localization of circATP2C1 and miR-654-3p was detected by fluorescence in situ hybridization (FISH) in PC3 and LNCaP cells. (**E**) In PC3 and LNCaP (**a**–**d**) cells, the CCK-8 assay was used to detect the effects of miR-654-3p and circATP2C1 on cell proliferation. (**F**) In PC3 and LNCaP cells, the Transwell assay was performed to assess the effects of miR-654-3p overexpression (**a**) or knockdown (**b**), combined with circATP2C1 overexpression or knockdown, on cell migration and invasion. (**G**) In PC3 and LNCaP cells, wound healing assays (scratch assays) were performed to assess the effects of miR-654-3p overexpression (**a**) or knockdown (**b**), combined with circATP2C1 overexpression or knockdown, on cell migration. NC, negative control; miR-654-3pmimics, miR-654-3p overexpression; miR-654-3pinhibitor miR-654-3 knockdown; si-circATP2C1, circATP2C1 knockdown; OE-circATP2C1, circATP2C1 overexpression. * *p* < 0.05, ** *p* < 0.01, **** *p* < 0.0001.

As circRNAs can sponge miRNAs as ceRNAs, potential target miRNAs of circATP2C1 were identified using the Circbank, Circbase, Starbase, and Circinteractome databases. It was found that miR-654-3p was the target miRNA of circATP2C1 (Figure 4A). Meanwhile, AGO2-RIP assay demonstrated that AGO2 could capture both miR-654-3p and circATP2C1 in PC3 and LNCaP cells (Figure 4B). Moreover, results of the luciferase reporter assay revealed that transfection of the miR-654-3p mimic decreased luciferase activity in cells containing the wild-type (WT) binding site of miR-654-3p in circATP2C1, whereas luciferase activity remained unaffected when cells were transfected with the miR-654-3p mimic in the presence of the mutant (MT) binding site of miR-654-3p in circATP2C1 (Figure 4C). Furthermore, the FISH assay showed co-localization of miR-654-3p and circATP2C1 in the cytoplasm of prostate cancer cells (Figure 4D). These data suggest that circATP2C1 sponges miR-654-3p in prostate cancer cells.

Subsequently, to understand the functional role of circATP2C1-regulated miR-654-3p in prostate cancer cells, its effects were evaluated. Results indicated that overexpression of miR-654-3p by mimics inhibited, whereas silencing of miR-654-3p by inhibitors promoted proliferation (Figure 4(Ea,d)), migration (Figure 4(Fa,b) and Figure 4(Ga,b)), and invasion (Figure 4(Fa,b)) of PC3 and LNCaP cells.

#### 3.1.4. CircATP2C1 Inhibits Ferroptosis to Drive Prostate Cancer Cell Proliferation, Migration, and Invasion

**Figure 5:**
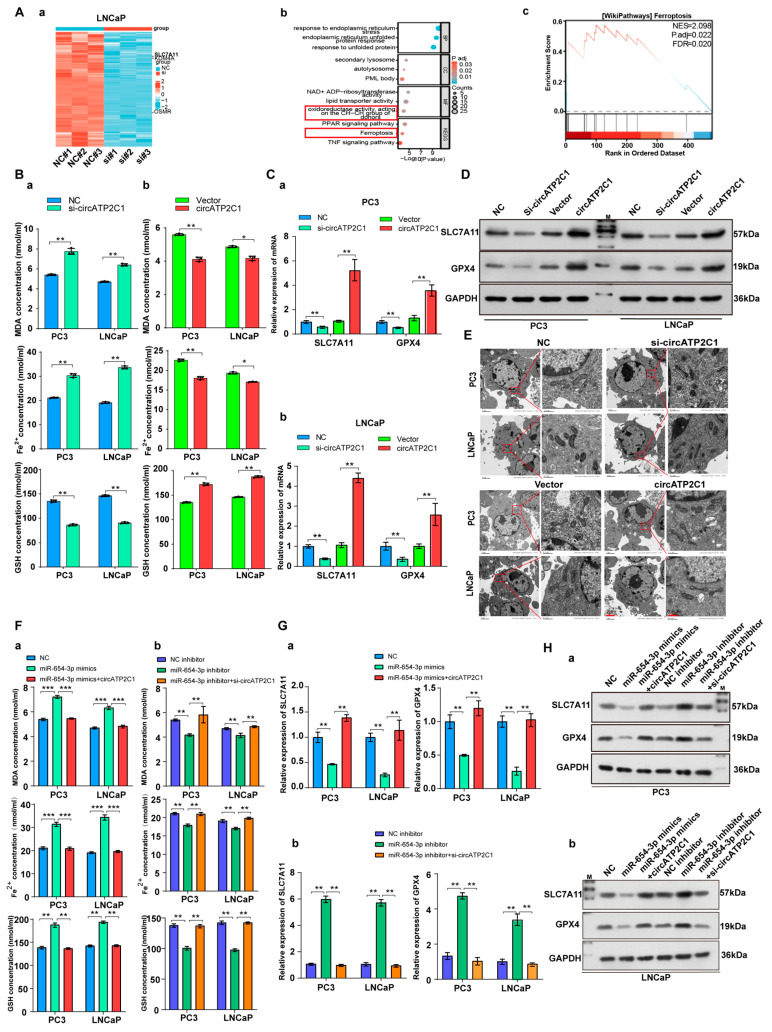
CircATP2C1 inhibits ferroptosis to facilitate prostate cancer cell proliferation, migration, and invasion. (**A**) Heatmap showing differentially expressed genes (DEGs) in LNCaP prostate cancer cells with knockdown of circATP2C1 (**a**). GO and KEGG analysis of differentially downregulated genes (**b**), and GSEA of enrichment of these downregulated genes in the ferroptosis pathway (**c**). (**B**) The effect of circATP2C1 knockdown (**a**) or overexpression (**b**) on MDA, ferrous ion (Fe^2+^), and GSH levels in PC3 and LNCaP cells was measured by ELISA. (**C**) qPCR detection of SLC7A11 and GPX4 expression in PC3 (**a**) and LNCaP (**b**) cells after treatment with circATP2C1 knockdown or overexpression. (**D**) Western blot (WB) analysis of SLC7A11 and GPX4 expression following circATP2C1 overexpression or knockdown in PC3 and LNCaP cells. (**E**) The effect of circATP2C1 overexpression or knockdown on mitochondrial morphology was examined by electron microscopy in PC3 and LNCaP cells. (**F**) In PC3 and LNCaP cells, the effects of miR-654-3p overexpression (**a**) or knockdown (**b**), combined with circATP2C1 overexpression or knockdown, on MDA, ferrous ion (Fe^2+^), and GSH levels were measured by ELISA. (**G**) qPCR analysis in PC3 and LNCaP cells assessed the effects of miR-654-3p overexpression (**a**) or knockdown (**b**), combined with circATP2C1 overexpression or knockdown, on GPX4 and SLC7A11 expression. (**H**) Western blot analysis in PC3 and LNCaP cells evaluated the effects of miR-654-3p overexpression (**a**) or knockdown (**b**), combined with circATP2C1 overexpression or knockdown, on GPX4 and SLC7A11 expression. NC, negative control; miR-654-3pmimics, miR-654-3p overexpression; miR-654-3pinhibitor miR-654-3 knockdown; si-circATP2C1, circATP2C1 knockdown; OE-circATP2C1, circATP2C1 overexpression; si-SLC7A11, SLC7A11 silence; OE-SLC7A11, SLC7A11 overexpression. * *p* < 0.05, ** *p* < 0.01, *** *p* < 0.001.

Next, to investigate the mechanism by which circATP2C1 regulates prostate cancer cells, RNA sequencing was performed in control and circATP2C1-knockdown LNCaP cells, and differentially expressed genes (DEGs) were identified (Figure 5(Aa)). The five most downregulated DEGs (Table 2) in circATP2C1-knockdown LNCaP cells are present in Table S2. Furthermore, GO and KEGG analyses of downregulated genes in circATP2C1-knockdown LNCaP cells showed that circATP2C1 is involved in oxidoreductase activity acting on the CH-CH group of donors, as well as in ferroptosis signaling pathways (Figure 5(Ab)). GSEA also revealed that the downregulated genes in circATP2C1-knockdown LNCaP cells were enriched in the ferroptosis signaling pathway (Figure 5(Ac)). To verify the effect of circATP2C1 on ferroptosis in prostate cancer cells, ELISA was performed to detect the levels of ferroptosis-related indicators MDA, Fe^2+^, and GSH in PC3 and LNCaP cells. The results indicated that circATP2C1 overexpression decreased the levels of MDA and Fe^2+^, while increasing the level of GSH in PC3 and LNCaP cells. In contrast, knockdown of circATP2C1 elevated the levels of MDA and Fe^2+^ but reduced the level of GSH in these cells (Figure 5(Ba,b)). SLC7A11 mRNA and protein levels in PC3 and LNCaP cells with circATP2C1 overexpression or knockdown were detected by qPCR assay and Western blot (WB). Results showed that circATP2C1 expression correlated positively with SLC7A11 expression in PC3 and LNCaP cells (Figure 5C,D). Downregulation of SLC7A11 can indirectly inhibit GPX4 activity by impairing the cystine uptake pathway, leading to a reduction in intracellular cystine levels and depletion of GSH biosynthesis. This decrease in GPX4 activity results in the accumulation of lipid peroxides and ultimately induces ferroptosis. Therefore, GPX4 expression was also detected by qPCR assay and Western blot (WB), and results indicated that circATP2C1 expression correlated positively with GPX4 expression in PC3 and LNCaP cells (Figure 5C,D). Furthermore, electron microscopy analysis revealed that circATP2C1 knockdown disrupted mitochondrial structure in PC3 and LNCaP cells (Figure 5E). Moreover, miR-654-3p overexpression decreased the levels of GSH and increased the levels of MDA as well as Fe^2+^ in PC3 and LNCaP cells (Figure 5(Fa,b). Conversely, miR-654-3p overexpression reduced SLC7A11 and GPX4 mRNA and protein levels in these cells (Figure 5G,H). However, circATP2C1 overexpression reversed the effects of miR-654-3p overexpression. Targetscan, miRwalk, and Starbase were used to predict the target genes of miR-654-3p, and the prediction results were jointly analyzed with the downregulated genes in circATP2C1-knockdown LNCaP cells. Three target genes were identified, including SLC7A11, lysine demethylase 4A (KDM4A), and oncostatin M receptor (OSMR) (Figure S1A). Results of the luciferase reporter assay indicated that transfection with miR-654-3p mimic reduced luciferase activity in cells containing the wild-type (WT) binding site of miR-654-3p in the SLC7A11 mRNA 3’UTR, whereas luciferase activity remained unaffected when cells were transfected with miR-654-3p mimic in the presence of the mutant (MT) binding site of miR-654-3p in the SLC7A11 mRNA 3’UTR (Figure S1B). Next, we investigated the role of SLC7A11 in prostate cancer cells. Initially, SLC7A11 was silenced by transfection with siRNA, and the SLC7A11 siRNA1 with higher knockdown efficiency was used for subsequent experiments. Additionally, SLC7A11 overexpression was achieved by transfection with the SLC7A11 expression vector (Figure S1C). Subsequently, whether miR-654-3p exerts effects on prostate cancer cells through SLC7A11 was investigated. SLC7A11 overexpression increased the proliferation (Figure S1(Da–d)), migration, invasion (Figure S1(Ea,b), and scratch assay (Figure S1(Fa,b)) of PC3 and LNCaP cells; conversely, miR-654-3p overexpression reversed the effects of SLC7A11 overexpression. Meanwhile, miR-654-3p knockdown decreased the levels of MDA and Fe^2+^, and increased GSH in PC3 and LNCaP cells (Figure S1(Ga,b)); conversely, miR-654-3p overexpression reduced levels of GSH. Moreover, miR-654-3p knockdown elevated SLC7A11 and GPX4 mRNA and protein levels in these cells (Figure S1H,I). However, SLC7A11 overexpression reversed the effects of miR-654-3p overexpression.

**Table 2:** Most downregulated DEGs in circATP2C1-knockdown LNCaP cells.

**Gene**	**Up/Down**	**Log2** **(Fold Change)**
PLIN1	Down	−5.41792616
AQP7	Down	−4.2309028
SLC7A11	Down	−4.027071623
AKR1C1	Down	−3.971601484
NR5A2	Down	−3.584328375

Here, circATP2C1 knockdown abolished the effects of miR-654-3p knockdown on proliferation, invasion, migration, and ferroptosis of PC3 and LNCaP cells. Taken together, these results suggest that circATP2C1 enhances proliferation, migration, and invasion by suppressing ferroptosis through sponging miR-654-3p to liberate SLC7A11 in prostate cancer cells.

#### 3.1.5. CircATP2C1 Promotes Proliferation, Migration, and Invasion by Suppressing Ferroptosis via Increasing SLC7A11 Expression in Prostate Cancer Cells

**Figure 6:**
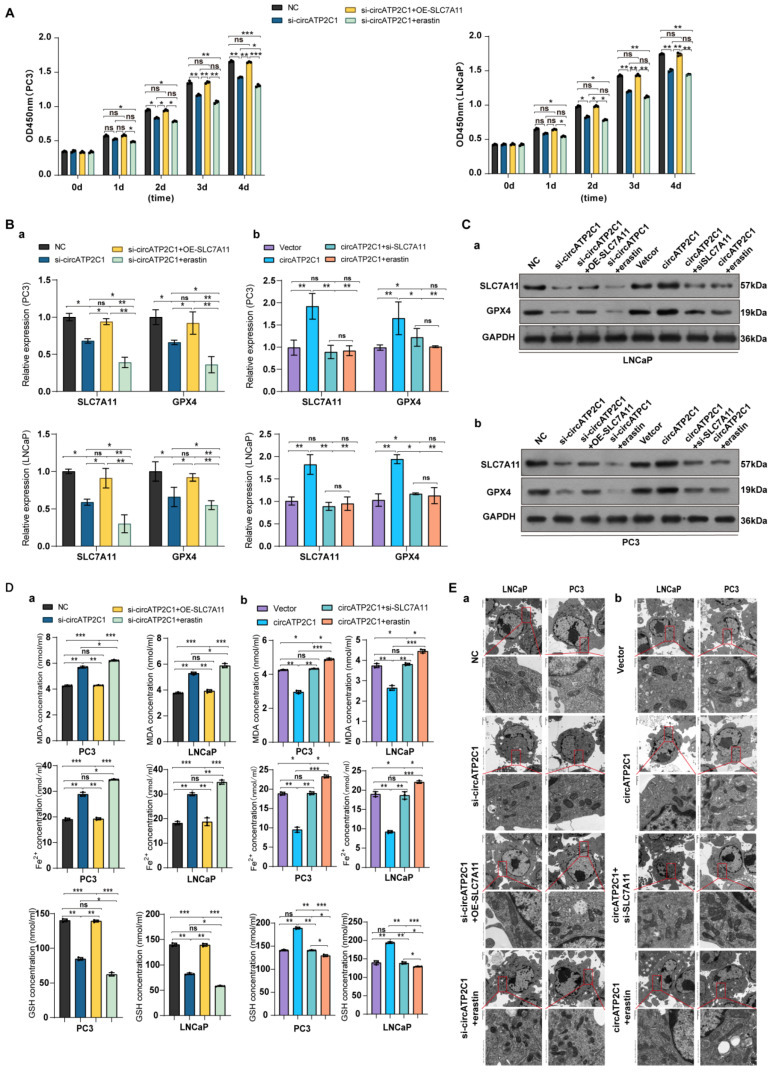
CircATP2C1 enhances proliferation, migration, and invasion by prohibiting ferroptosis via elevating SLC7A11 expression in prostate cancer cells. (**A**) Effect of circATP2C1 knockdown combined with SLC7A11 overexpression or erastin on cell proliferation detected by Cell Counting Kit-8 (CCK-8) assay in PC3 and LNCaP cells. (**B**) Effects of circATP2C1 knockdown combined with SLC7A11 overexpression or erastin (**a**), and circATP2C1 overexpression combined with SLC7A11 knockdown or erastin (**b**) on GPX4 and SLC7A11 expression detected by quantitative PCR (qPCR) in PC3 and LNCaP cells. (**C**) Effects of circATP2C1 knockdown combined with SLC7A11 overexpression or erastin (**a**), and circATP2C1 overexpression combined with SLC7A11 knockdown or erastin (**b**) on GPX4 and SLC7A11 expression detected by Western blot (WB) in PC3 and LNCaP cells. (**D**) Effects of circATP2C1 knockdown combined with SLC7A11 overexpression or erastin (**a**), and circATP2C1 overexpression combined with SLC7A11 knockdown or erastin (**b**) on malondialdehyde (MDA), Fe^2+^, and glutathione (GSH) levels detected by ELISA in PC3 and LNCaP cells. (**E**) Effects of circATP2C1 knockdown combined with SLC7A11 overexpression or erastin (**a**), and circATP2C1 overexpression combined with SLC7A11 knockdown or erastin (**b**) on mitochondria, as detected by electron microscopy in PC3 and LNCaP cells. NC, negative control; si-circATP2C1, circATP2C1 knockdown; OE-circATP2C1, circATP2C1 overexpression; si-SLC7A11, SLC7A11 silence; OE-SLC7A11, SLC7A11 overexpression. * *p* < 0.05, ** *p* < 0.01, *** *p* < 0.001, ns = not significant.

It was found that overexpression of SLC7A11 accelerated the proliferation, migration, and invasion of circATP2C1-knockdown PC3 and LNCaP cells, but the ferroptosis inducer erastin inhibited the proliferation (Figure 6A), invasion, and migration (Figure S2 (Ba) ) of circATP2C1-knockdown PC3 and LNCaP cells. Moreover, both silencing of SLC7A11 and erastin suppressed the proliferation (Figure S2A), migration, and invasion (Figure S2 (Bb)) of circATP2C1-overexpressed PC3 and LNCaP cells. Furthermore, SLC7A11 overexpression increased, but erastin reduced, SLC7A11 and GPX4 levels in circATP2C1-knockdown PC3 and LNCaP cells (Figure 6 (Ba,Ca)). In contrast, both silencing of SLC7A11 and erastin decreased SLC7A11 and GPX4 levels in circATP2C1-overexpressed PC3 and LNCaP cells (Figure 6 (Bb,Cb)). By contrast, SLC7A11 overexpression decreased, but erastin increased, as did MDA and Fe^2+^, and elevated GSH levels in circATP2C1-knockdown PC3 and LNCaP cells (Figure 6 (Da)). Meanwhile, both silencing of SLC7A11 and erastin elevated MDA and Fe^2+^, and decreased GSH levels in circATP2C1-overexpressed PC3 and LNCaP cells (Figure 6(Db)). Additionally, electron microscopy analysis showed that overexpression of SLC7A11 reversed, yet erastin disrupted the mitochondrial structure in circATP2C1-knockdown PC3 and LNCaP cells (Figure 6 (Ea)). Both silencing of SLC7A11 and erastin aggravated the disruption of mitochondrial structure in circATP2C1-overexpressed PC3 and LNCaP cells (Figure 6 (Eb)). The above results suggest that circATP2C1 enhances proliferation, migration, and invasion by inhibiting ferroptosis through upregulation of SLC7A11 expression in prostate cancer cells.

#### 3.1.6. CircATP2C1 Promotes the Tumorigenicity of Prostate Cancer by Inhibiting Ferroptosis In Vivo

**Figure 7:**
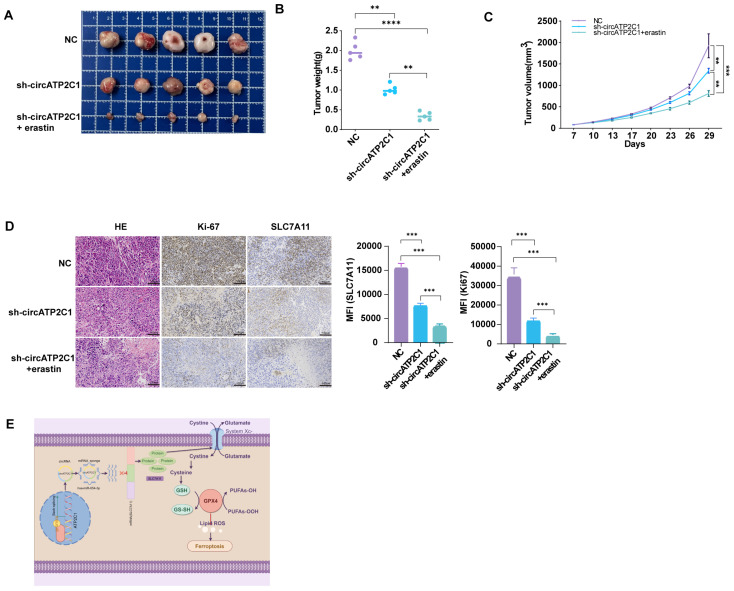
CircATP2C1 accelerates the tumorigenicity of prostate cancer by suppressing ferroptosis in vivo. (**A**) Photographs demonstrate the inhibitory effects of the ferroptosis inhibitor erastin on the growth of transplanted tumors in nude mice, using the PC3 prostate cancer cell line with circATP2C1 knockdown. (**B**) The bar graph illustrates the combined effects of circATP2C1 inhibition and erastin treatment on the weight of transplanted tumors. (**C**) The bar graph illustrates the combined effects of circATP2C1 inhibition and erastin treatment on the volume of transplanted tumors. (**D**) Immunohistochemical staining of tumor tissue sections from nude mice using the Ki-67 and SLC7A11 antibody. (**E**) Schematic diagram illustrating the regulatory mechanisms identified in this study. NC, negative control; sh-circATP2C1, circATP2C1 knockdown. ** *p* < 0.01, *** *p* < 0.001, **** *p* < 0.0001.

To further determine the oncogenic role of circATP2C1 in vivo, we constructed a xenograft tumor mouse model by subcutaneously injecting PC3 cells into the right dorsal flanks of nude mice. The results showed that circATP2C1 knockdown reduced the size and weight of tumors derived from PC3 cells. Injection of erastin, a ferroptosis inducer, further enhanced the effects of circATP2C1 knockdown on tumor development (Figure 7A–C). Furthermore, circATP2C1 knockdown decreased SLC7A11 expression in tumors derived from PC3 cells, and injection of erastin improved the effect of circATP2C1 knockdown on SLC7A11 expression (Figure 7D). These results suggest that circATP2C1 accelerates the tumorigenicity of prostate cancer by suppressing ferroptosis through upregulation of SLC7A11 expression in vivo. See the schematic diagram of regulatory mechanisms for this study (Figure 7E).

## 4. Discussion

This study demonstrates that circATP2C1 drives proliferation, migration, and invasion by suppressing ferroptosis. It does so by increasing SLC7A11 expression through acting as a molecular sponge for miR-654-3p in prostate cancer cells. circATP2C1 was first found to regulate prostate cancer proliferation and metastasis. Although the role of circRNAs in cancer has been under scrutiny for many years, a variety of circRNAs have been identified as potential markers. circRNAs have a stable structure and resist degradation; therefore, circATP2C1 is also expected to be a stable marker for prostate cancer.

miR-654-3p is expressed at low levels in a variety of cancers, and it can effectively inhibit the proliferation and metastasis of non-small cell lung cancer [29]. Meanwhile, miR-654-3p serves as a prognostic marker for hepatocellular carcinoma and inhibits the proliferation, migration, and invasion of cancer cells [30]. Downregulation of miR-654-3p is associated with poor prognosis and promotes the proliferation and migration of colon cancer cells [31]. Our findings indicate that miR-654-3p is expressed at low levels in prostate cancer. Furthermore, miR-654-3p induces ferroptosis to suppress prostate cancer cell proliferation and migration. Therefore, miR-654-3p may serve as a nucleic acid-based therapeutic agent, as well as a diagnostic and prognostic marker for prostate cancer.

As a member of the solute carrier family, SLC7A11 encodes the light chain subunit of the cystine/glutamate transporter Xc−system, also known as xCT. SLC7A11 promotes glutathione synthesis by mediating cystine uptake and glutamate release, protects cells from oxidative stress, and maintains cellular oxidation–reduction homeostasis. The SLC7A11/GPX4 signaling axis is a central mechanism for the suppression of ferroptosis. SLC7A11 is overexpressed in a variety of malignant tumors, such as breast [32], ovarian [33], hepatocellular [34], and lung cancer [35]. SLC7A11 is closely related to tumor growth, prognosis, metastasis, and treatment of malignant tumors, and its high expression is an unfavorable factor for the disease-specific survival rate of eight types of tumors, including prostate cancer, adrenocortical carcinoma, bladder cancer, and squamous cell carcinoma of the head and neck [36]. Studies of the association of SLC7A11 with the development, progression, and treatment of prostate cancer are at an early stage. It has been found that SLC7A11 is involved in the development of resistance to docetaxel in prostate cancer, and the transcription factor AP-2γ (TFAP2C) inhibits ferroptosis in prostate cancer through the c-Myc/miR-25-3p/SLC7A11 signaling axis, which in turn promotes its resistance to chemotherapy [37,38]. Furthermore, this study reveals that SLC7A11 is highly expressed in prostate cancer; the SLC7A11/GPX4 signaling pathway regulates cellular ferroptosis, thereby influencing prostate cancer proliferation and metastasis. Consequently, SLC7A11 may serve as a potential diagnostic and prognostic marker for prostate cancer, as well as an effective therapeutic target.

Our findings reveal that circATP2C1 increases SLC7A11 expression by sponging miR-654-3p, as miR-654-3p targets SLC7A11 in prostate cancer cells. A previous study has reported that miR-654-3p targets cAMP-responsive element binding protein 1 (CREB1) to repress presenilin-1 (PSEN1) transcription in sinonasal squamous cell carcinoma [39]. Additionally, CREB1 upregulates SLC7A11 expression in lung adenocarcinoma [40]. Except for directly targeting SLC7A11, miR-654-3p might suppress SLC7A11 transcription by targeting CREB1 in prostate cancer cells.

## 5. Conclusions

In summary, the discovery of the circATP2C1/miR-654-3p/SLC7A11 signaling pathway has enabled us to understand that circRNAs can regulate the proliferation and metastasis of prostate cancer cells by modulating ferroptosis. This finding provides new targets and insights for treating prostate cancer.

## Data Availability

The datasets used and analyzed during the current study are available from the corresponding authors on reasonable request. Circbank (http://www.circbank.cn (accessed on 25 September 2024)). Circbase (https://circbase.org/ (accessed on 25 September 2024)). Starbase (https://rnasysu.com/encori/index.php (accessed on 25 September 2024)). Circinteractome (https://circinteractome.nia.nih.gov/ (accessed on 25 September 2024)). GEO datasets (GSE179321 and GSE155792, https://www.ncbi.nlm.nih.gov/geo (accessed on 25 September 2024)). Targetscan (https://www.targetscan.org/vert_71/ (accessed on 25 September 2024)). miRwalk (http://mirwalk.umm.uni-heidelberg.de/ (accessed on 25 September 2024)).

## Supplementary Materials

The following supporting information can be downloaded at: https://www.mdpi.com/article/10.3390/cancers17213571/s1, Figure S1: miR-654-3p inhibits proliferation, migration and invasion by triggering ferroptosis via SLC7A11 in prostate cancer cells; Figure S2: CircATP2C1 enhances proliferation, migration and invasion by prohibiting ferroptosis via elevating SLC7A11 expression in prostate cancer cells; Table S1: Clinicopathologic characteristics of the human prostate cancer tissue microarray; Table S2: The  downregulated DEGs in circATP2C1-knockdown LNCaP cells.

## Abbreviations

AGO2	Argonaute 2
AGO2-RIP	AGO2-RNA immunoprecipitation
ANT	Adjacent non-tumor tissue
CCK-8	Cell Counting Kit-8
CeRNA	Competitive endogenous RNA
CircRNA	Circular RNA
DEGs	Differentially expressed genes
ELISA	Enzyme-linked immunosorbent assay
FBS	Fetal bovine serum
FISH	Fluorescence in situ hybridization
GEO	Gene Expression Omnibus
GO	Gene Ontology
GPX4	Glutathione Peroxidase 4
GSEA	Gene Set Enrichment Analysis
GSH	Glutathione
HCAR1	Hydroxy-carboxylic acid receptor 1
IHC	Immunohistochemistry
KDM4A	Lysine demethylase 4A
KEGG	Kyoto Encyclopedia of Genes and Genomes
LNM	Lymph node metastasis
MCT1	Monocarboxylate transporter 1
MDA	Malondialdehyde
MT	Mutant
NC	Negative control
NCBI	National Center for Biotechnology Information
OD	Optical density
OSMR	Oncostatin M receptor
PFA	Paraformaldehyde
qRT-PCR	Quantitative reverse transcription-PCR
SCD1	Stearoyl coenzyme A desaturase 1
SD	Standard deviation
shRNA	Short hairpin RNA
siRNA	Small interfering RNA
SLC7A11	Solute carrier family 7 member 11
SREBP1	Sterol regulatory element binding protein 1
TFAP2C	Transcription factor AP-2γ
WB	Western blot
WT	Wild-type
