# Supplementary material for: CircATP2C1 Drives Prostate Cancer Progression Through miR-654-3p-Mediated SLC7A11 Upregulation and Ferroptosis Suppression
Source: Cancers (Basel). 2025 Nov 5;17(21):3571. doi: 10.3390/cancers17213571 (PMC12609431; doi:10.3390/cancers17213571)
Supplement: Supplementary file 1 [file cancers-17-03571-s001.zip › cancers-3903945-Supplementary.pdf]

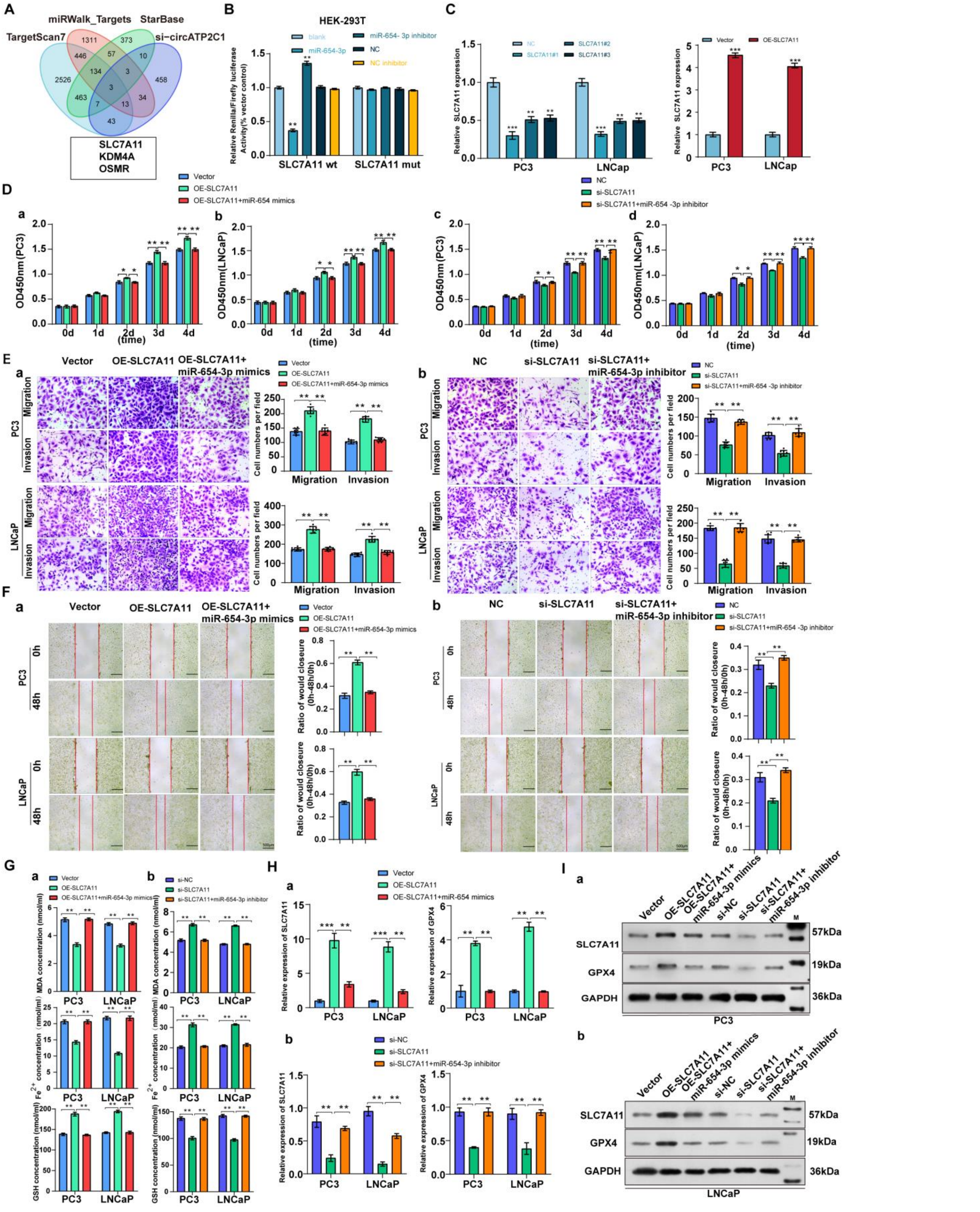

Figure S1. miR-654-3p inhibits proliferation, migration and invasion by triggering ferroptosis via SLC7A11 in prostate cancer cells.A Bioinformatics analysis of the overlap between target genes of miR-654-3p and downregulated genes in circATP2C1-knockdown in LNCaP cells. B Dual-luciferase reporter assay demonstrating miR-654-3p binding to the SLC7A11 3'UTR in 293T cell. C qPCR analysis of the effects of SLC7A11 overexpression or knockdown in PC3 and LNCaP cells.D In PC3 (a

and c) and LNCaP (b and d) cells, a CCK8 assay was used to assess the effects of SLC7A11 and miR-654-3p by overexpression or knockdown on cell proliferation. E In PC3 and LNCaP cells, a Transwell assay was used to evaluate the effects of SLC7A11 overexpression (a) or knockdown (b), combined with miR-654-3p overexpression or knockdown, on cell migration and invasion. F In PC3 and LNCaP cells, a wound healing (scratch) assay was performed to assess the effects of SLC7A11 overexpression (a) or knockdown (b), together with miR-654-3p overexpression or knockdown, on cell migration. G In PC3 and LNCaP cells, ELISA was used to measure the effects of SLC7A11 overexpression (a) or knockdown (b), plus miR-654-3p overexpression or knockdown, on MDA, Fe^{2+}, and GSH content. H In PC3 and LNCaP cells, qPCR was performed to determine the effects of SLC7A11 overexpression (a) or knockdown (b), along with miR-654-3p overexpression or knockdown, on the expression of GPX4 and SLC7A11. I In PC3 and LNCaP cells, Western blot (WB) analysis was conducted to evaluate the effects of SLC7A11 overexpression (a) or knockdown (b), combined with miR-654-3p overexpression or knockdown, on the expression of GPX4 and SLC7A11.

NC, negative control;miR-654-3pmimics,miR-654-3p overexpression;miR-654-3pinhibitor miR-654-3 knockdown;si-SLC7A11, SLC7A11 silence;OE-SLC7A11, SLC7A11 overexpression. *P<0.05, **P<0.01, ***P<0.001.

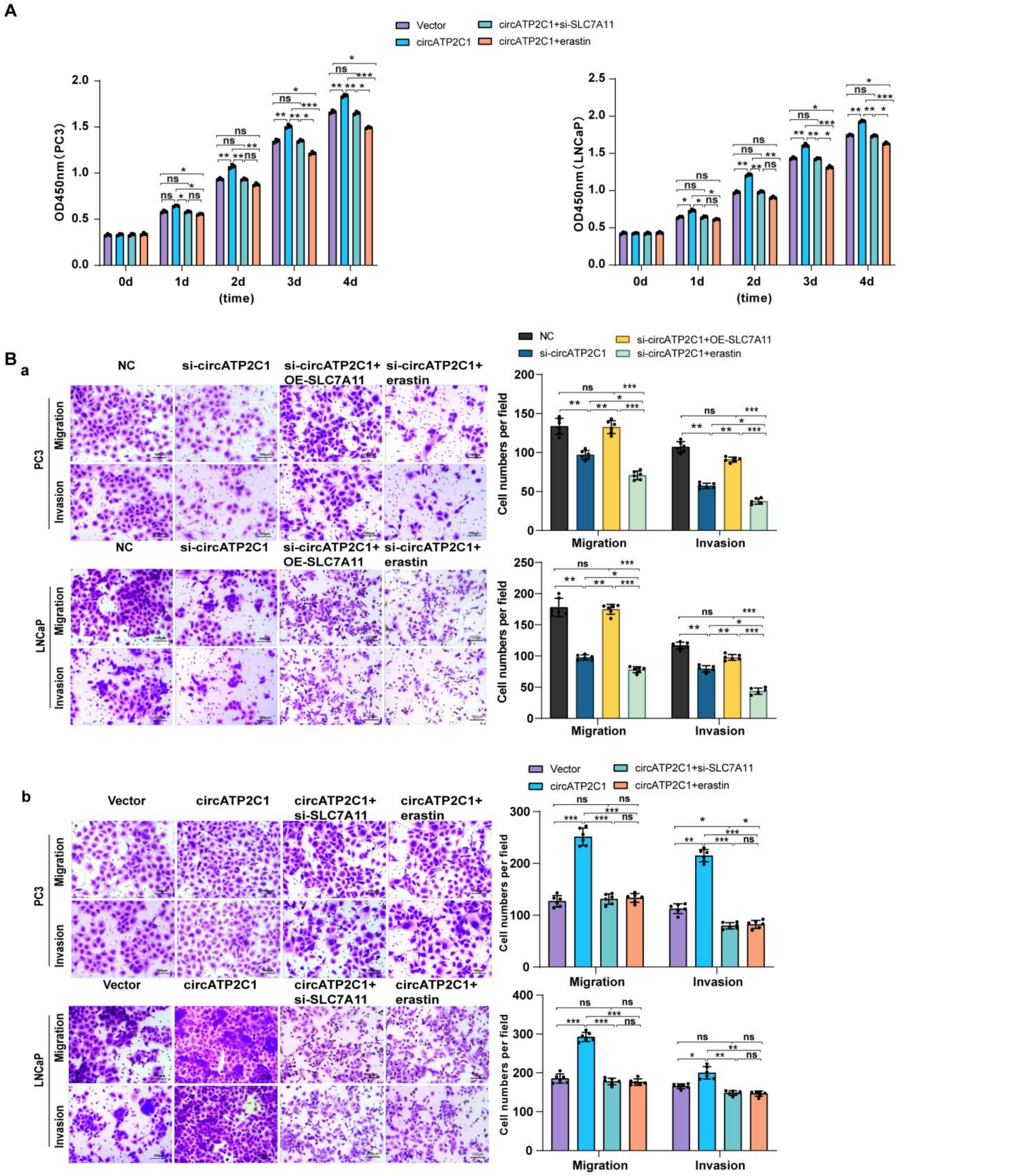

Figure S2. CircATP2C1 enhances proliferation, migration and invasion by prohibiting ferroptosis via elevating SLC7A11 expression in prostate cancer cells.A Effect of circATP2C1 overexpression combined with SLC7A11 knockdown or erastin on cell proliferation detected by Cell Counting Kit-8 (CCK-8) assay in PC3 and LNCaP cells.B Effects of circATP2C1 knockdown combined with SLC7A11 overexpression or erastin (a), and circATP2C1 overexpression combined with SLC7A11 knockdown or erastin (b) on cell migration and invasion detected by Transwell and scratch assays in PC3 and LNCaP cells.NC, negative control; si-circATP2C1, circATP2C1 knockdown; OE-circATP2C1, circATP2C1 overexpression; si-SLC7A11, SLC7A11 silence; OE-SLC7A11, SLC7A11 overexpression. *P<0.05, **P<0.01, ***P<0.001.
