# Supplementary figures and images for: CircATP2C1 Drives Prostate Cancer Progression Through miR-654-3p-Mediated SLC7A11 Upregulation and Ferroptosis Suppression
Source: Cancers (Basel). 2025 Nov 5;17(21):3571. doi: 10.3390/cancers17213571 (PMC12609431; doi:10.3390/cancers17213571)

Figure 5D

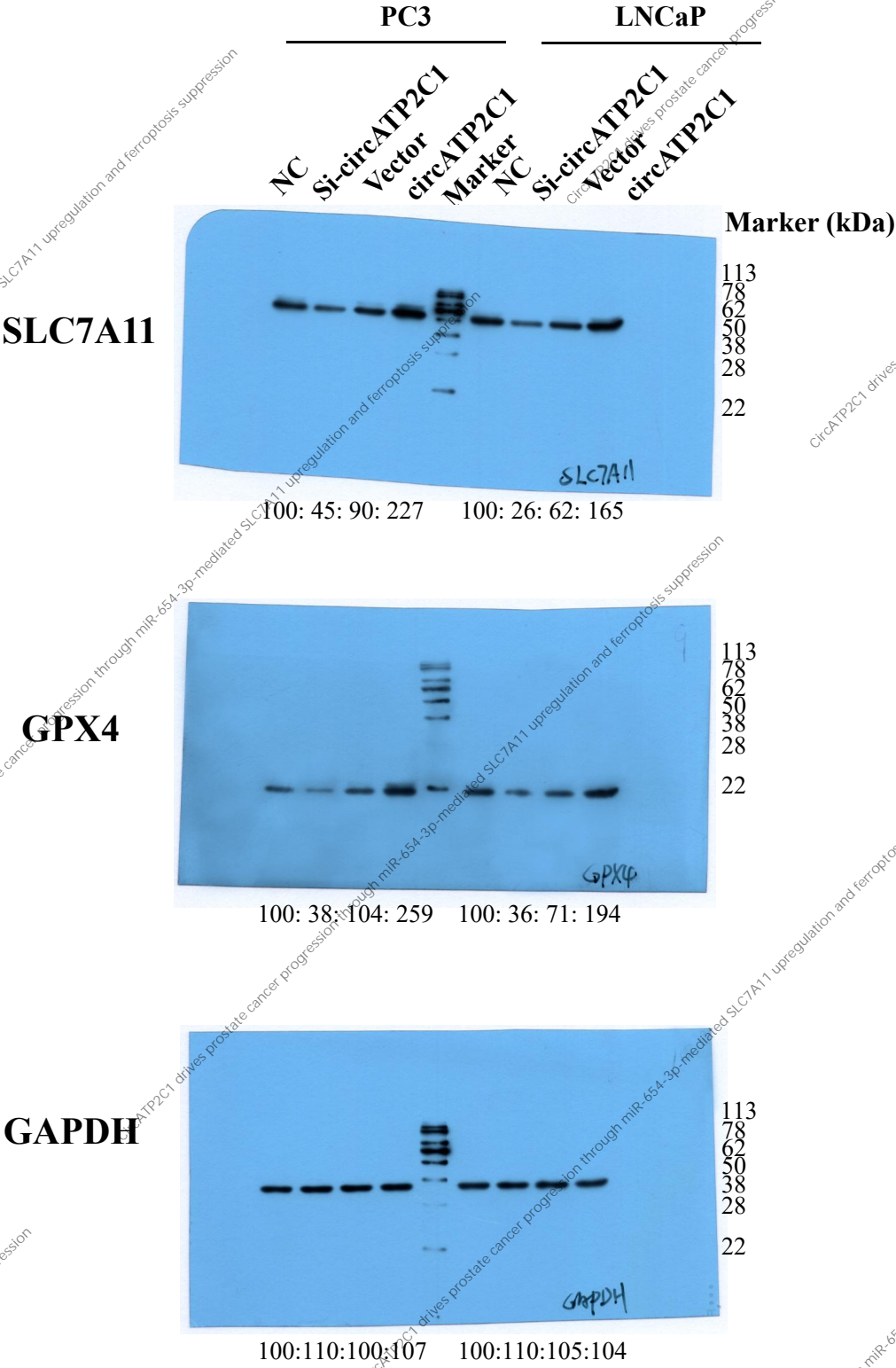

Figure 5H

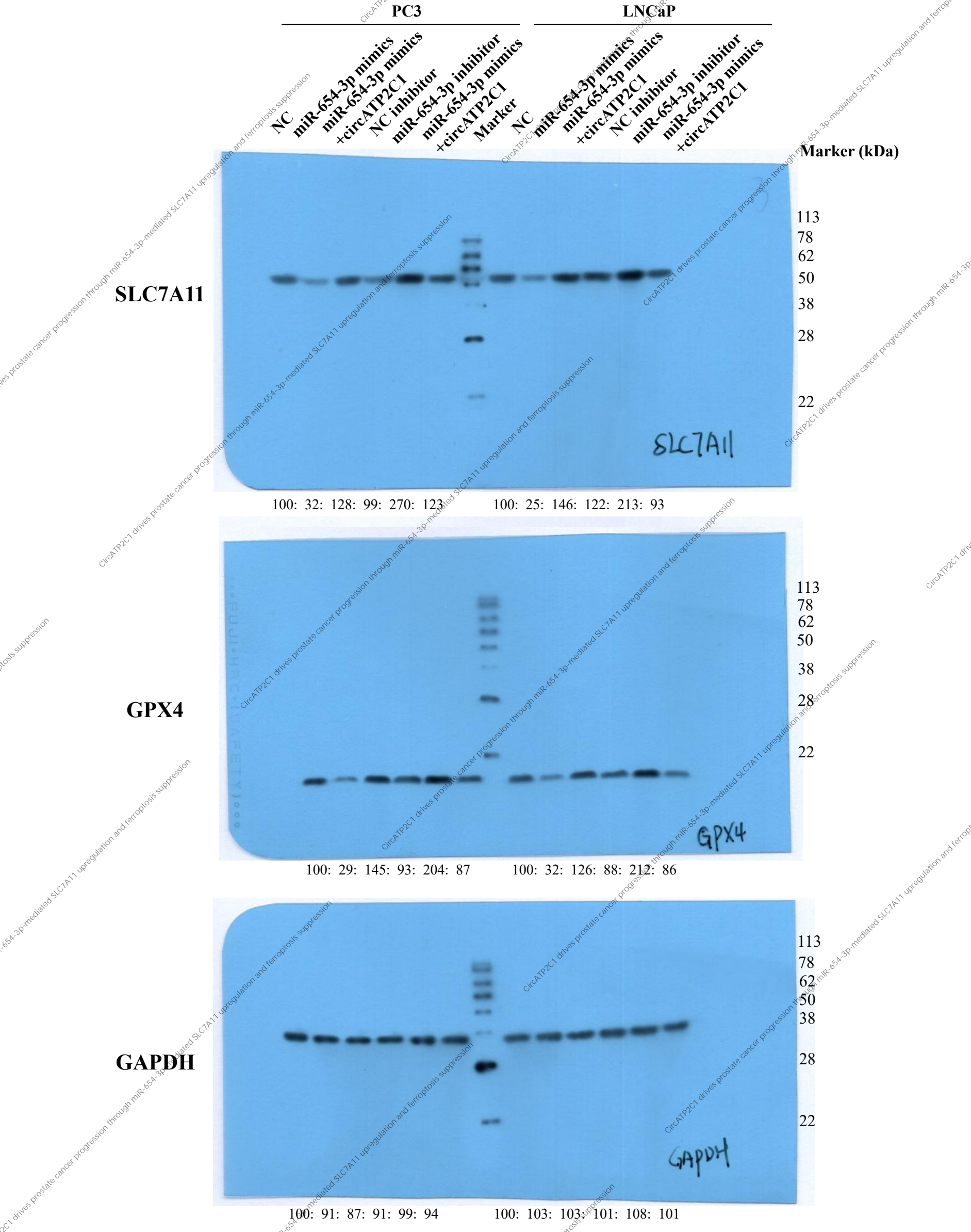

Figure 6C-a

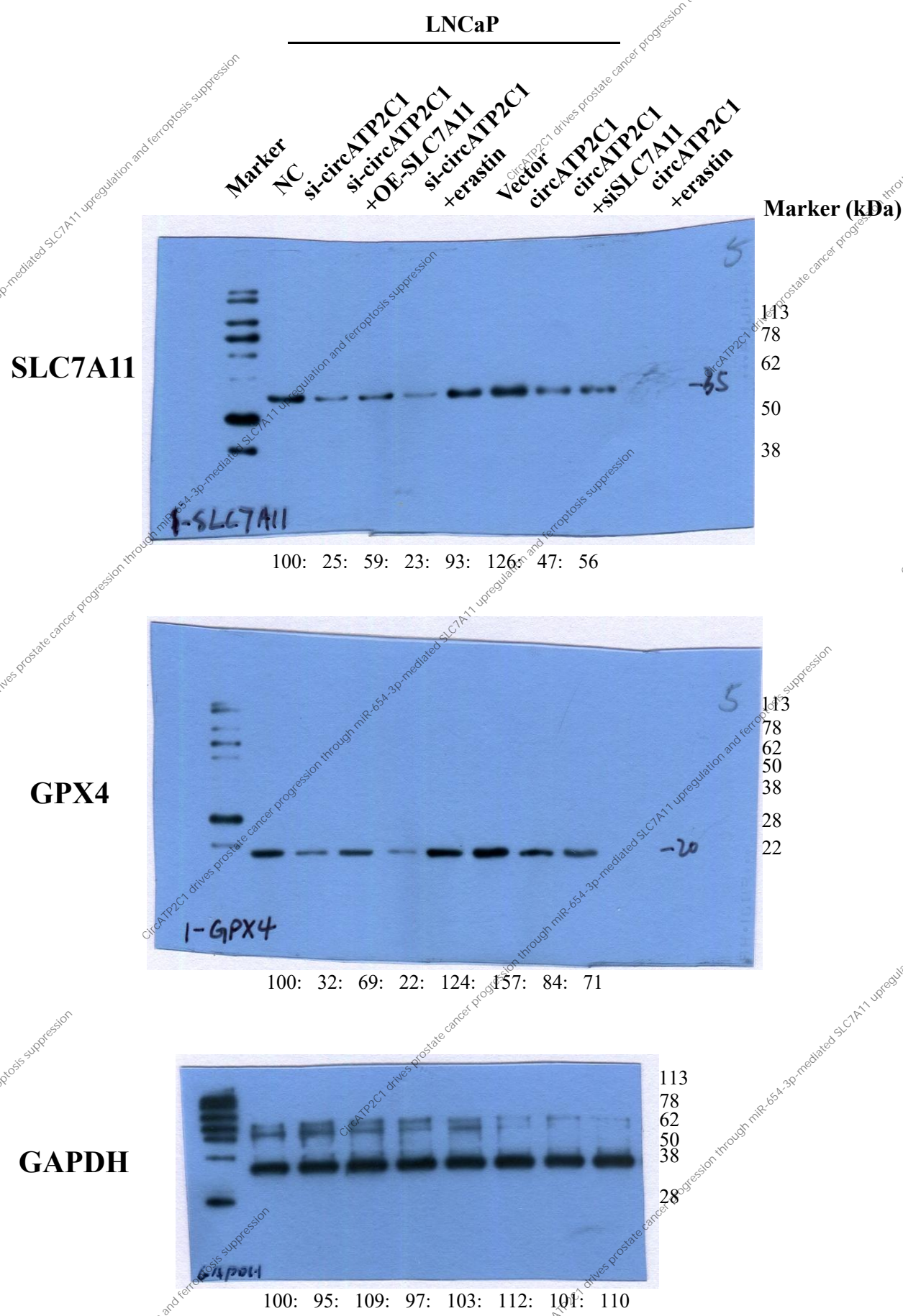

Figure 6C-b

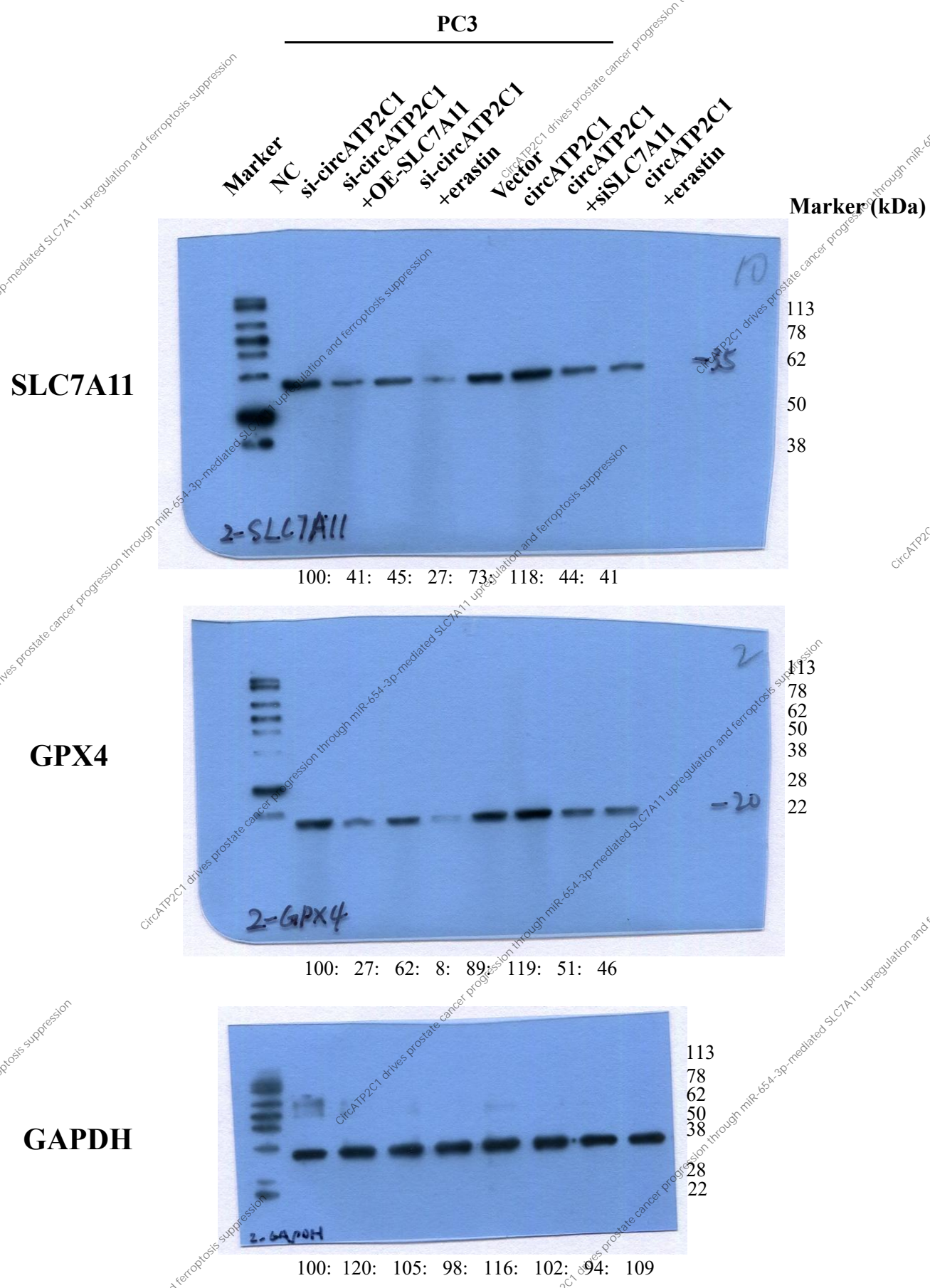

Figure S1I-a

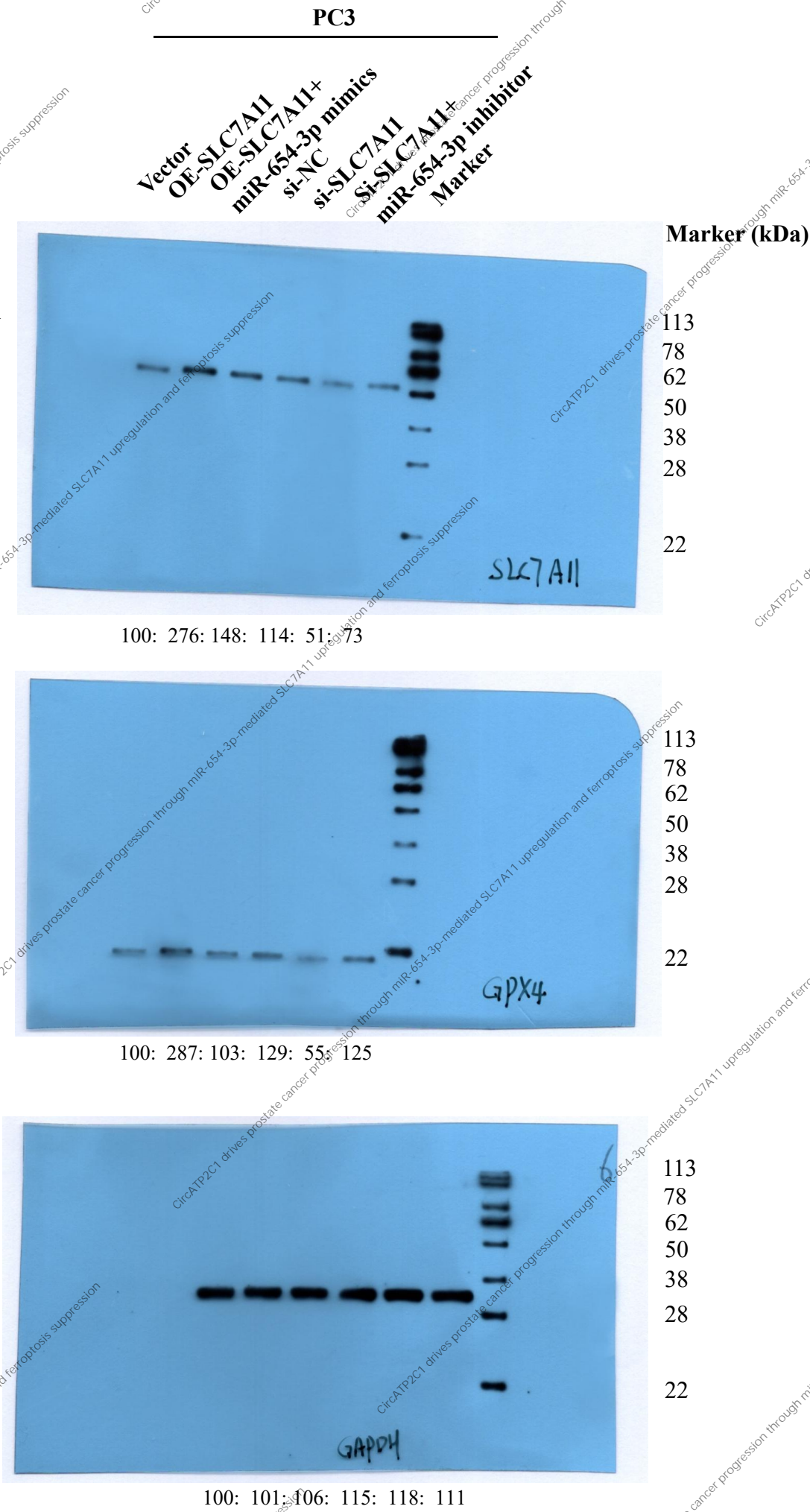

Figure S1I-b

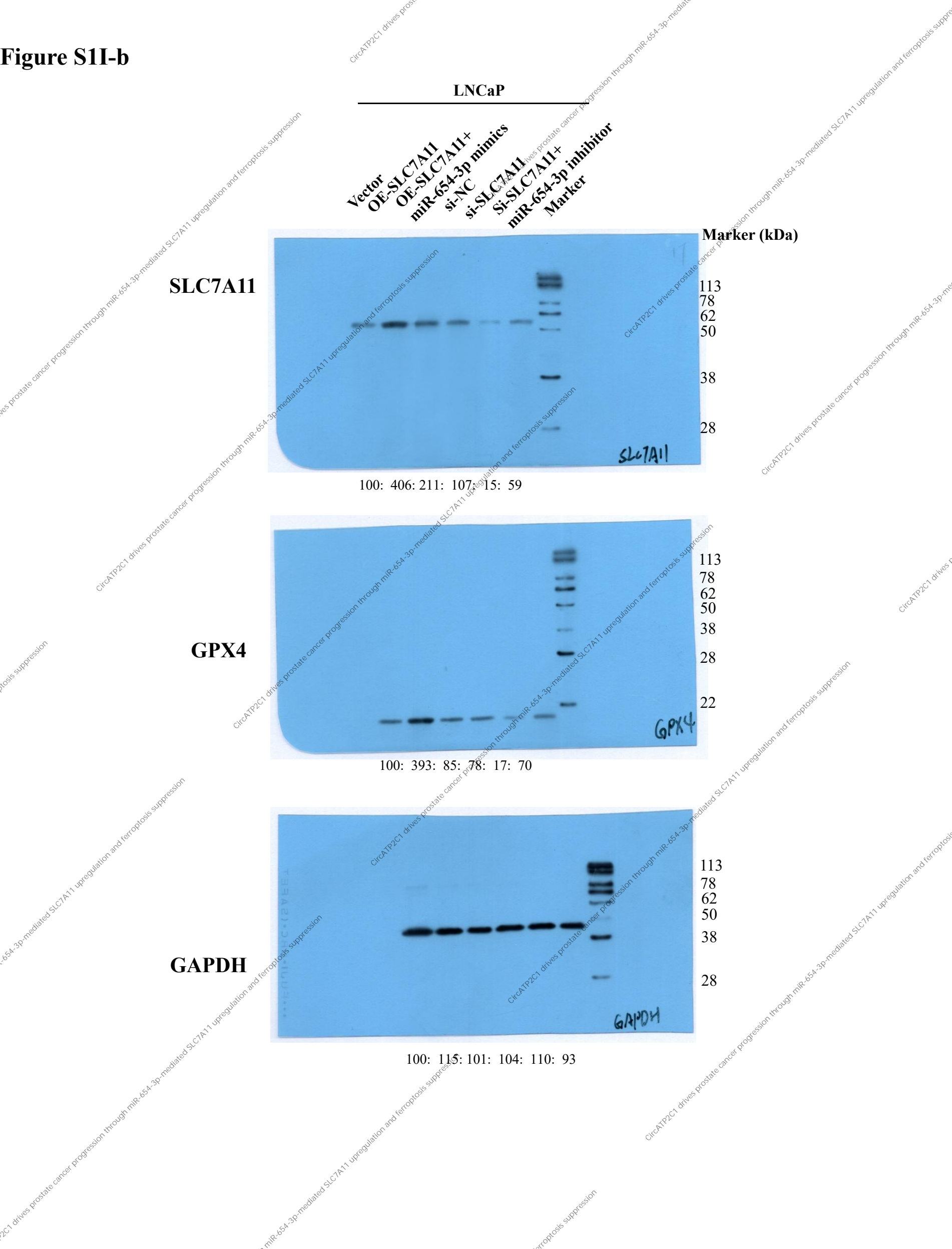

Supplement: Supplementary file 1 [file cancers-17-03571-s001.zip › cancers-3903945-Original Images for Blots and Gelsú¿the original Western blot figuresú⌐.pdf]
